# Practical Medicine

**Published:** 1876-12

**Authors:** 


### II. PRACTICAL MEDICINE.

1. *Rheumatism Treated with Salicylate of Soda.* PROF. A. CLARKE. (*The Med. Record*, Oct. 14, 1876.)

Prof. C. treated eleven cases of acute rheumatism—all that occurred in his ward of Bellevue from April 1 to June 1—with this drug. In nine of the cases there was early improvement following the use of the medicine. In two cases the amelioration was more gradual. The influence of the medicine in "lowering the fever heat and diminishing the excited pulse were as marked as its power to relieve pain."

The formula used in all the cases is as follows:

℞ Acid salicylic ---------------------------------- ʒ iij

Sodæ bicarbonat ---------------------------------- ʒ ij

Glycerine. } *aa* --------------------------------- ℥ ij

Aq. }

M. Sig. Tablespoonful every two hours for the first day, and afterward the same dose six times a day.

No unpleasant effect of any kind was noticed after the administration of the medicine.

2. *A Case of Idiopathic Glossitis.* WINGATE. (*Boston M. and S. Jour*, Sept. 7, 1876.)

The patient was a robust Irish girl, twenty years old. The inflammation seemed causeless. The tongue became enormously swollen, hard and covered with a yellow coating; pain was caused on attempting to protrude it; saliva flowed constantly from the mouth and to speak intelligibly was impossible. She had a moderate fever (103.2° F.) and a pulse of 130. There was nausea and vomiting at first; there was constipation, and, later great weakness.

The invasion of the disorder was gradual; it remained at a high point of severity for three days and slowly subsided. As improvement became established the coating disappeared from the tongue, leaving a dark red surface.

3. *The Cause of Chorea.* BAKER. (*Detroit Review*, Nov., 1876.)

Dr. B. first quotes from Prof. Stevens, of Albany, who holds that chorea is due to an over taxing of the powers of accommodation of the eyes; an over tiring, and, hence, exhaustion of the ciliary muscle in the adjustment of the focus of the eyes for different distances.

In hypermetropia, as objects approach the eyes, the function of accommodation is more severely taxed than in normal eyes. Normal eyes are always at rest when accommodated for distance; hypermetropic eyes are never at rest except when closed.

Effort at accommodation and at convergence are simultaneous and proportioned to each other. "Normal eyes accommodated for twelve inches are also converged for twelve inches." "In hypermetropia the balance is lost. Accommodation for twelve inches requires an exertion that in the normal eye would accommodate for—say six inches. The eyes would then be converged for six inches. So; there would be accommodation for a foot and convergence for only half that distance. Confusion of nervous and muscular action results.

"Young children have their eyes almost always accommodated for near objects." In these early years the ciliary muscle is kept in a state of extreme tension constantly. When there happens to be hypermetropia there is a conflict constantly between the muscles of accommodation and of convergence, unless there is a compromise by which one eye "agrees to a constant squint." The constant fatigue aud irritation from the conflict referred to, occurring in sensitive children, often at a time when they are over taxed at school, is such as strongly to predispose to—even to cause chorea, as Dr. S. believes.

Dr. B. believes, with Dr. S., that strain of the ciliary muscle may cause chorea, but that long continued muscular strain of any sort is competent to lead to the disorder, as well as that of the muscle referred to. Over exertion and tiring of any muscle easily lead to tremors. A sudden fright or severe exertion may make the tremors persistent, and we have chorea. Dr. B. quotes a number of cases to substantiate his position.

4. *Maternal Impressions.* WADDEL. (*Detroit Review*, Nov., 1876.)

After a long and exhaustive examination of opinions and authorities as to the anatomical and physiological relations of the mother to the embryo, as to the seeming proof that the mother's mind has power to change the conformation of the fœtus in utero, and to cause deformity; and after considering such evidence in the light of modern embryology and pathological anatomy, Dr. W. ably sums up his conclusions substantially as follows:

1. During embryonic existence certain parts may be hindered or arrested in their development, while others are not disturbed.

2. Ectropic viscera of the abdomen, spina bifida, cleft palate, hare-lip, webbed fingers and toes, etc., are only evidence of arrested developement of embryonic, abdominal, spinal and maxillary processes, or, in case of the webbed extremities, the continuation of the embryonic hand or foot of the second month.

3. Any agency causing arrest of development of any portion of the fœtus must necessarily operate prior to the evolution of that part.

4. The cause of arrested development may be local or general; (a) injuries to the mother's abdomen; (b) diseases of the uterus or its membranes; (c) diseased ovum from diathesis of either parent; (d) hereditary transmission of deformity.

5. Excessive development of parts of the fœtus may obtain, resulting in nevi, aneurisms by anastomoses, supernumerary fingers, toes, etc.

6. Such peculiarities are, in common with family resemblances, frequently transmitted through generations.

7. Intra-uterine amputations are the result of amniotic bands, placental adhesions, fracture, or from pressure of a loop of the umbilical cord.

8. Amniotic bands or placental adhesions may result from inflammation of the uterus, its decidua, or inflammatory disease of the fœtus.

9. These false membranes may cause amputations of extremities, which may afterward be absorbed as may the false membranes.

10. The so-called double monsters, or fœtus *in fœtu*, are the result of double cicatricula on the blasto-dermic membrane of a single ovum.

11. Twins with a common chorion also result from the development of double cicatricula on the blasto-dermic membrane of a single ovum.

12. In either case there is always unity of sex.

13. The nearness of the primitive traces to each other, determines whether impregnation will result in separate twins or double monster.

14. In twins with single chorion or anastomosis of the placental vessels, one fœtus may become perfectly developed while the other may become monstrous.

15. The development of the abnormality in such cases depends on local anatomical causes, and is governed by definite laws.

16. Every known form of malformation in man has its analogue in the lower animals, birds, fishes and reptiles.

The author next puts the inquiry whether maternal mental impressions may arrest the development of the fœtus in utero and cause deformity; or cause disease in the uterus or membranes, resulting in false bands or placental adhesions; or cause the umbilical cord to encircle and amputate a limb, or cause the death of the fœtus; whether such impression can reach and act on the newly impregnated ovum, leading the double cicatricula to approach each other so closely as to result in union and double monstrosity; or can destroy or deform one fœtus in utero, leaving another in the same membranes uninjured?

Many congenital deformities being shown to arise from causes unconnected with maternal influence, he asks, "Is it possible that at another time exactly the same deformity is produced by the maternal impressions?"

"When it is remembered that no nervous connection exists between the embryo and mother; that there is no direct blood communication; that the mother's mind can have no influence in causing the pathological states which have been shown to be the cause of malformation; that during the first weeks of fœtal life the ovum is surrounded by anatomical conditions precluding maternal influences, whereas it has been shown that the vast majority of malformations have their origin in that period of embryonic life in which the ovum is still homogeneous blastema; when all these facts are remembered it cannot," he says, "be believed that the mother's mind can change the conformation of the fœtus *in utero*."

5. *Epileptic Phenomena in a Case of Cerebral Tumor.* DeBonis. (*Monthly Abst.*, Oct., 1876.)

In an article on the *Annali Clinici dello Ospedale Incurabili di Napoli*, anno i. fasc. 2, Dr. DeB. reviews the recent doctrines regarding epilepsy, and accepts vascular spasm as an explanation; in short, he recognizes epilepsy as a vaso-motor necrosis of the brain. He relates the case of a man, aged sixty, who first suffered severe pains in the frontal and parietal regions, extending to the occiput; the pain was intermittent. Convulsions afterwards appeared; they were preceded by noises in the ears and vertigo, and were repeated several times a day. When young he had had gonorrhœa, soft ulcers, and syphilides. From the anamnesis and the symptoms observed during the attack, the diagnosis was made of epilepsy with chronic meningitis from cerebral syphilis, probably with gummata disseminated in the anterior and middle lobes of the right hemisphere, and encephalitis affecting the origin of the seventh pair. Most of the symptoms—paleness, dilatation of the pupils, clonic convulsions, etc.—were principally manifested on the right side. At the necropsy, there were found congestion of the membranes of the brain, chronic meningitis, and seven gummatous masses in the cortical substance toward the upper part and the base of the cerebral lobes, some being of old, others of recent formation. There was no appreciable lesion of the medulla oblongata, pons Varolii, cerebral peduncles, hippocampus, etc.

Dr. DeB. remarks that this case shows that from the cerebral mass there may arise irritations, which are directed along the converging fibres to the medulla oblongata and peduncles, determining a super-excitation of the vaso-motor centres, whence arises spastic contraction of the vessels, as shown by the pallidity of face and loss of consciousness. He thus explains the convulsions by spastic ischæmia of the nervous centers.—*Lond. Med. Record*, Aug. 15, 1876.

6. *The Diagnosis of Auditory Vertigo.* GOWERS. (*Monthly Abstract*, Oct., 1876.)

Dr. G. read a paper on this subject before the Section on Medicine, British Medical Association. He said the form of vertigo which depends on an affection of the organ of hearing has very little apparent connection with its cause, and frequently has so obtrusive an association with the *gastric* functions that the real nature of these cases is constantly misconceived by the sufferers, and frequently by our own profession. There are several ways in which the stomach symptoms obscure the real nature of the affection. The paroxysms of vertigo commonly lead to vomiting. Sometimes they cause only an attack of dyspepsia, to which the giddiness is thought to be due. But, further, so intimate are the mutual action and reaction of the pneumogastric and equilibrium nerves and centers, that in the subjects of " auditory vertigo " a paroxysm, quite special in form, may be excited by a primary gastric disturbance, to which the whole trouble is naturally ascribed. The subjects of auditory vertigo, besides their acute paroxysms, are usually liable to slighter but more continuous sense of disturbed equilibrium, taking the form not of a definite movement, but merely of vague instability, and this is in constant relation to the gastric function. The slightest stomach disturbance at once excites the feeling, and careful dietetic management is necessary to keep the patient free from it. Among the points to be especially attended to in diagnosing auditory from pure gastric vertigo are the following: The occurrence of intense paroxysms of vertigo, especially if repeated, is in favor of their labyrinthine origin. In many forms of vertigo, paroxysms occur, but in none so intense as in this form. The character of the sensation is of great significance. When purely gastric, it is usually indefinite; and labyrinthine vertigo is usually definite. There is a sense of movement in a certain direction, usually uniform. Often there is actual movement. Lastly, evidence is usually to be obtained of affection of the auditory nerve or apparatus—unilateral, or greater on one side than on the other—tinnitus and deafness. The deafness may be due to atmospheric vibrations, or to those conducted through the skull. The latter, a well known symptom of labyrinthine disease, is of great service in the diagnosis of auditory vertigo. Two illustrative cases were narrated. In the first, characteristic auditory vertigo co-existed with some other affection of cranial nerves, interference with smell and taste, etc. The patient suffered also from chronic ulcer of the stomach, and the gastric disturbance excited paroxysms of vertigo. The patient always fell to the right and backwards, and the right ear alone presented perverted function. Although the special gastric association in this case was accidental, the confusion in diagnosis to which it gave rise was typical, and was only cleared by careful investigation of the form of the vertigo. In the second case, a gentleman aged thirty-five, suffered from slight dizziness, with dyspeptic symptoms, to which he ascribed it. On examination, however, there was clear evidence of labyrinthine mischief, and a history of paroxysms of vertigo most characteristic in form. In these he fell to the right, the auditory affection being on the left side. He fell with such violence as to knock down a friend who was on his right side. In other attacks objects seemed to move from the right, although he did not fall. One severe paroxysm was excited by a hearty meal after long fasting. The paper concluded with some remarks on the diagnosis of the severer paroxysms from slight apoplectic siezures and from attacks of *petit mal.—British Med. Journal*, Aug. 26, 1876.

7. *The Nursing and Weaning of Infants and Young Animals.* MAGNE. (*L'Union Méd.*, No. 118.)

The difference between the nursing of the young of human beings and other animals is neither explicable by their organization nor conformable to the laws of physiology. Why, for example, is an artificial diet generally considered so injurious to infants, when it is so successful in young animals; and why do alimentary substances, when substituted for or added to milk, prove injurious to the former and useful to the latter?

The writer believes it to be irrational to give an infant breast milk exclusively till it is one year old, and afterward to administer to it pap containing bread, rice, milk, etc. These latter are eminently unfit to be substitutes for milk, and yet milk itself is insufficient as a sole aliment for children who have passed the sixth, seventh, eighth and tenth month. These statements are based upon the ascertained inefficacy of those articles of food in supplying the material for tissue waste, and in repairing the losses which the organism is liable to suffer.

The needs of the infant increase with its age. As its skeleton becomes longer and its muscles more voluminous, it requires more phosphates and albuminoid matters than during the first months of life. Now, the mother's milk cannot, for twelve months, augment in the same proportion as the requirements and according to the needs of her nursling. The writer has, on several occasions, recommended for children eleven and twelve months of age, of fair constitution, but who were yet unable to walk, the eating of raw eggs. One or two of these, daily, sufficed to produce a clear color of the skin, firm flesh and the ability to walk unaided.

Those who busy themselves with the rearing of the young of animals have brought about successive and considerable modifications in their system, and have decided that incomparably the best results are obtained by early weaning. Without an artificial diet, commenced almost immediately after birth, the public would never see at the Expositions immense bulls with cylindrical bodies and thick muscles, and powerful draught horses which gain the prizes. The more intelligent of stock raisers employ a nutritious substance whose composition is nearly that of milk, but which is much more preferable because it is less watery and more substantial.

The more complicated the composition of one of these milk substitutes the more it is nutritious. Products which naturally serve as nourishment are thought to be much more available by extracting their essence, by disengaging their alimentary part. This is an error.

Meat is too systematically excluded from the dietary of the nursery. Man is carnivorous in infancy as in adult life. The extract and juice of meat cannot take the place of meat itself for the child. Among available articles mentioned are, eggs, raw and cooked; meat, and the flour of various vegetable productions.

The discussion of this paper evoked great opposition among the members of the Academy of Medicine.

